# Office Visitors

**Published:** 1906-11

**Authors:** 


### OFFICE VISITORS.

They met in our office reception room, one an old time gentleman, the other an elderly lady. Both faithful patients. The first by virtue of success in life and vigorous old age was used to having his say out when in conversation. The other a society lady rather gossipy and inclined to anticipate the trend of converse and take up the thread herself, scarcely allowing the other "a word in edgewise." Why how-de-do Mr. ———, I'm awfully glad to see you, I hope you are well and feeling much better than I expect to when I come out of that room, though I must say the doctor is very gentle and hurts just as little as he can, but you know every kind of work on your teeth hurts, even at the hands of the easiest dentist."

"The doctor didn't hurt me a bit," replied Mr. ——, "because—

"Yes, I know, because, I guess, you don't wear the kind that can be hurt."

"Yes, that's so, I've lost nearly all my natural ones and the doctor has fixed me up nicely with false ones and I can use—"

"Well, it is wonderful that at your age you have any at all of the natural set; why my father lost all of his a long time before he was as old as you, as for me—"

"If the dentists of forty or fifty years ago had the knowledge in treating and saving teeth that these of the present age have I never would have lost my teeth for they were pretty strong, but I neglected them. They were monstrous hard to pull and—

"Law me, I don't see how you could have stood it, and in those days they didn't have gas or cocaine, I would have—"

"They had ether and chloroform and whisky, but I didn't take either one, just stood it. One of them though nearly got the best of me. This dentist, I forget his name, that had an office—"

"Well, I'd taken anything or everything, why I couldn't have stood it. When mother had her's all out she took ether. Dr.

—— gave it to her; she couldn't stand it just so like you, so she sent—"

"Well, I didn't stand it, I sat it and—"

"Oh now, you know I didn't mean that you stood up to have—"

"Well, as I was a'telling you, I went to this fellow and he caught hold of my tooth and he pulled and he pulled until my head and neck, I reckon, was like a goose's at an old time goose-pulling, but—"

"My, but what is an old time goose-pulling? I never saw—"

"No, I reckon you never did. It was before your time. But as I was asaying, he pulled and he pulled, and he couldn't get it and he got so mad he just threw his instrument across the room under the sofa. I asked him if he was through and he—"

"Why I didn't know you was so good at making a pun and you stood—I mean sat—"

"I didn't make any pun and he didn't make any fun for me. I got up and walked—"

"You did make the pun if you didn't have any fun. There now, I'm a poetess if you are a punster, he! he! he!"

"Well, I can laugh over the recollection of it now, but there wasn't any poetry about it then for me. Then shortly before Dr. —— sold out to Dr. ——. I went to him to have t tooth pulled and I guess he was drinking and—"

"Yes, he used to keep pretty full all the time before he left A—— and, do you know, they tell me he used to treat his wife shamefully. He actually on one—"

"Hold on, and let me tell you about the tooth. He got out his forceps and jabbed down on a tooth and broke it to pieces, square off, even with the gums. By G—, ses he, d—n if I havn't pulled the wrong tooth. But do you know it cured the ache in the other one."

"Well if that wasn't the strangest thing. It's equal to blowing into one eye to get a cinder out of the—"

"And do you know, that fellow came to me—had the cheek to come to me—for a recommend when he was going to move away, and I gave it to him. Afterwards I went—"

"That was mighty good of you. I wouldn't have done it; just to think of his drinking and butchering you. I would have—"

"Yes, I went to his successor and he got it out in a half dozen pieces and—"

"Why he must have broken it all to pieces. The idea of—"

"That's just what he did, but I must be going. Good by. I mighty glad to have seen you."

"Good by. You know he didn't treat his wife well, and poor thing she died and he married again, but they tell me she's leading him a dance and—well good by. Excuse me, doctor I am coming right now. I've had such a nice little chat with dear old Mr. ——. He is a dear old man. I hope I haven't kept you waiting.'

